# Treatment of Coronary Perforation in a Bifurcation: Two‐Stent Strategies With Fenestrated Covered Stents

**DOI:** 10.1002/ccd.70450

**Published:** 2025-12-29

**Authors:** Jakob U. Lindner, Janine Pöss, Holger Thiele, Dmitry S. Sulimov

**Affiliations:** ^1^ Department of Internal Medicine/Cardiology Heart Center Leipzig at Leipzig University and Leipzig Heart Science Leipzig Germany; ^2^ Department of Internal Medicine/Cardiology German Army Hospital Ulm Ulm Germany

**Keywords:** bifurcation, coronary perforation, covered stents, percutaneous coronary intervention (PCI)

## Abstract

Coronary perforations are a dreaded complication for any interventional cardiologist. The treatment of this complication is exceptionally difficult when it occurs in a coronary bifurcation. While a conservative approach or a balloon occlusion might be sufficient in some cases, additional treatment is sometimes necessary. Here, we present two cases, where complex two‐stent strategies with covered stents were necessary to successfully treat the bleeding. The aim is to discuss the feasibility of this method, while also analyzing published cases where fenestration of a covered coronary stent was successfully performed. The commonly used material is discussed, including its capabilities and limitations. With the proper knowledge about the different treatment options and the required material readily available, this difficult complication can be successfully treated in the catheterization laboratory.

**Abbreviations**
CABG: coronary artery bypass grafting
CCS: Canadian Cardiovascular Society
CP: coronary perforation
CTO: chronic total occlusion
D1: first diagonal branch
DES: drug eluting stent
EBU: extra back‐up catheter
LAD: left anterior descending artery
LCX: left circumflex artery
LM: left main coronary artery
NSTEMI: non‐ST‐elevation myocardial infarction
PCI: percutaneous coronary intervention
RCA: right coronary artery
SCAI: Society for Cardiovascular Angiography & Interventions
TAP: T and small protrusion
TIMI: Thrombolysis in Myocardial Infarction
vaECMO: veno‐arterial extracorporeal membrane oxygenation

## 1 Introduction

Coronary perforations (CP) are rare complications and, depending on their severity and location, may be treated with prolonged balloon occlusion, embolization and/or covered stents. Implantation of covered stents over the orifice of side branches leads to occlusion of the branches and may result in clinically significant type 4a myocardial infarction.

A particularly challenging scenario is a perforation at a bifurcation involving a significant side branch that cannot be compromised. In this article, we present two cases with complex bifurcation treatment, in which a two‐stent strategy using covered stents was required to manage coronary perforation. In order to preserve coronary flow, fenestration of the stent‐membranes had to be performed. The feasibility of the fenestration has been demonstrated before by Taniguchi et al. and Werner et al. for different graft types [1, 2].

## 2 Case Reports

### 2.1 Case 1

**Figure 1:**
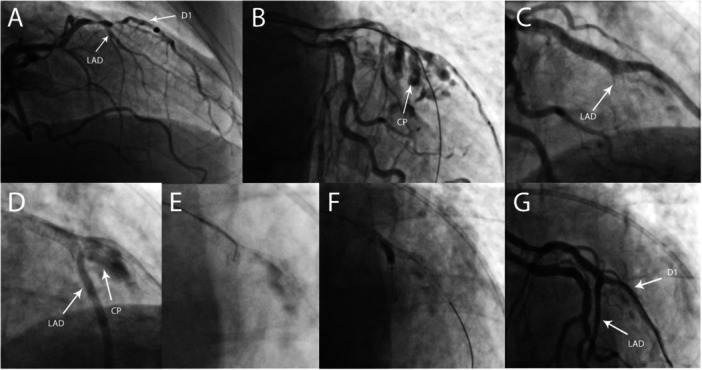
Case 1. TAP stenting of the LAD‐D1 bifurcation with covered stents. (A) Initial angiogram showing severe stenosis of the LAD and D1. (B) Angiogram after rotational atherectomy into the D1 with 1.25 mm and 1.5 mm burrs, wiring of the LAD and subsequent dilatation. The arrow indicates a large Ellis type 3 CP. (C) Angiogram after covered stent (Papyrus) implantation LAD‐D1. The arrow in indicates the antegrade obstructed flow to the LAD. (D) Despite obstructed antegrade flow in the LAD, an type II endoleak (arrow CP) can be seen through collateral flow to the LAD (arrow LAD). (E) Perforation of the covered stent membrane with a dual‐lumen microcatheter and a stiff guidewire (Progress 140). The previously implanted DES in the distal LAD was used to guide the membrane perforation. (F) Fenestration of the covered stent with a 3.0 × 12 mm semi‐compliant balloon. (G) Final result after TAP with another covered stent (Papyrus) in the mid‐LAD. No CP is visible and TIMI grade 3 flow was observed in all coronary arteries. CP, coronary perforation; D1, first diagonal branch; LAD, left anterior descending artery; TAP, T‐and‐protrusion; TIMI, Thrombolysis in Myocardial Infarction.

An 80‐year‐old male with unstable angina underwent invasive coronary angiography which showed a severely calcified 99% stenosis of the bifurcation of the left anterior descending artery (LAD) and the first diagonal branch (D1) (Figure 1A). Percutaneous coronary intervention (PCI) was performed via radial access with a 6 French (F) catheter. The distal LAD could not be wired because of severe calcification and angulation. The D1 was wired with a Fielder XT‐A (Asahi Intecc, Japan) but since the lesion was uncrossable, the decision was made to prepare it with rotational atherectomy. Subsequently, wire manipulations were easy, and the LAD could be wired successfully. Predilation of the D1 and LAD was performed at nominal pressure using 2.5 mm (D1) and 3.0 mm (LAD) ballons, demonstrating good balloon expansion. The following angiogram revealed a large Ellis type 3 perforation (Figure 1B); therefore, the proximal LAD was immediately occluded with a 3.0 × 20 mm balloon and pericardiocentesis was performed. Due to the complex anatomy of the bifurcation and severe bleeding, the exact region of the perforation could not be identified. A 2.5 × 20 mm Papyrus covered stent (Biotronik, Germany) was implanted in the D1 just distal to the ostium after unsuccessful prolonged balloon occlusion. However, due to ongoing bleeding, additional treatment of the bifurcation was deemed necessary.

A drug eluting stent (DES) (Xience Pro, Abbott Vascular, USA) was implanted in the LAD just distal of the D1 ostium to improve further navigation. Subsequently, a 3.0 × 15 mm Papyrus covered stent was implanted from the proximal LAD into the diagonal branch, overlapping the first covered stent and covering the mid‐LAD (Figure 1C). Due to good collateralization of the distal LAD, the bleeding from the bifurcation was still visible and could be classified as an endoleak type II, which required further action (Figure 1D). An attempt to coil this endoleak through a collateral artery failed, so the decision was made to use a two stent technique with covered stents. A dual lumen microcatheter (Sasuke, Asahi Intecc, Japan) was introduced into the bifurcation over the D1 wire and the Papyrus stent graft was perforated with a Progress 140 wire (Abbott vascular, USA) via the 2nd lumen of the microcatheter (Figure 1E). The previously implanted DES in the medial LAD was used as a reference for wire navigation. After fenestration of the Papyrus stent graft with a 3.0 × 12 mm balloon (Figure 1F) a 3rd covered stent was implanted into the LAD in T and protrusion (TAP)‐technique with a small protrusion into the bifurcation and final kissing‐balloon dilation was performed with two 2.5 mm balloons. Immediately after this step, no further bleeding was observed, and the procedure was completed with Thrombolysis in Myocardial Infarction (TIMI) grade 3 flow in all vessels (Figure 1G). The patient developed a type IV‐a myocardial infarction but remained stable and asymptomatic. The pericardial drainage was removed 18 h after the procedure and the patient was discharged 3 days later. At 2‐year follow‐up the patient presented with new onset mild stable angina (Canadian Cardiovascular Society [CCS] class 1) but refused further angiographic evaluation.

### 2.2 Case 2

**Figure 2:**
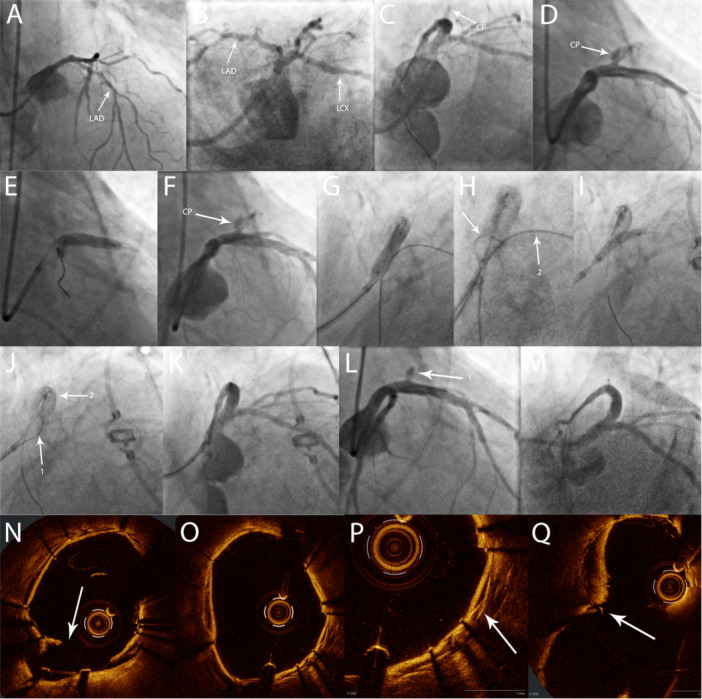
Case 2. Culotte stenting of the left main bifurcation with covered stents. (A)/(B) Initial angiogram showing severe stenosis of the LAD. (C) Angiogram after provisional stenting LM‐LAD and postdilation with OPN‐Balloon. The arrow indicates a small Ellis type 1 CP. (D) Angiogram after 24 h. The arrow indicates a large Ellis type 3 CP in the proximal LAD. (E) Implantation of a covered stent (Papyrus) in the proximal LAD. (F) Persistent Ellis Type 3 perforation after covered stent implantation (arrow). (G) Implantation of a covered stent (Papyrus) LM‐LAD, occluding the LCX Ostium. (H) Perforation of the covered stent membrane with a dual‐lumen microcatheter and stiff guidewire (Confianza Pro 12, Arrow 1), using the trapped guidewire in the LCX as reference (Arrow 2). (I) Implantation of a covered stent (Papyrus) LM‐LCX. (J) Perforation of the covered stent membrane with a dual‐lumen microcatheter and stiff guidewire (Confianza Pro 12, Arrow 1). The trapped guidewire and the previously implanted stent in the LAD were used as a reference (Arrow 2). (K)/(L) Final result after kissing‐balloon dilatation. Arrow 1 indicates a small, residual CP (Ellis Type 1). (M) Control angiogram after 48 h with no residual CP visible and a good result in the stents. (N) OCT after fenestration of the first covered stent. The arrow indicates the disruption in the membrane after balloon dilatation. (O)/(P) OCT in the LM after implantation of the second covered stent. The two overlapping membranes are clearly visible (arrow). (Q) OCT in the LM‐bifurcation at the end of the procedure. The arrow indicates a small tear in the membrane, due to overexpansion of the second covered stent. CP, coronary perforation; LAD, left anterior descending artery; LM, left main coronary artery; LCX, left circumflex coronary artery; OCT, optical coherence tomography. [Color figure can be viewed at wileyonlinelibrary.com]

A 78‐year‐old male patient presented with non‐ST‐elevation myocardial infarction (NSTEMI) and cardiogenic shock in Society for Cardiovascular Angiography & Interventions (SCAI) stage D. Coronary angiography showed a coronary two‐vessel disease with chronic total occlusion (CTO) of the RCA and a long, severely calcified lesion of the proximal and medial LAD (Figure 2A/B).

Due to refractory cardiogenic shock, a peripheral venoarterial extracorporeal membrane oxygenation (vaECMO) was established, before PCI of the proximal and mid LAD, which was considered to be the culprit lesion. The lesion was dilatable and a long DES was implanted in a provisional technique from the left main (LM) into the proximal LAD. Due to underexpansion of the stent, postdilation with a 3.5 mm OPN balloon (SIS medical, Switzerland) was deemed necessary. After the OPN balloon inflation, an Ellis Type 1 CP was identified and treated with prolonged balloon occlusion. After 11 min of balloon occlusion, the perforation crater remained visible; however, there was no connection to the pericardial space and no evidence of pericardial effusion (Figure 2–C). Since there was TIMI 3 flow in the main branches, the procedure was terminated and the patient was monitored on the intensive care unit. After 24 h, a large, hemodynamic relevant pericardial effusion was detected. An immediately performed coronary angiography revealed a severe Ellis Type 3 perforation in the proximal LAD (Figure 2D). After pericardial drainage, a 3.5 × 15 mm Papyrus covered stent was implanted in the proximal LAD (Figure 2E). Following postdilation, a significant residual perforation remained visible; therefore, the decision was made to extend the treatment to the LM bifurcation (Figure 2F). A Fielder XT wire was introduced into the LCX as a marker for the fenestration. Subsequently, an additional 4.0 × 15 mm Papyrus stent was implanted from the LM into the LAD with overlap to the previously implanted Papyrus (Figure 2G). Next, a Sasuke dual lumen microcatheter was introduced over the LAD wire in the LM. After 3 attempts, the membrane of the Papyrus was successfully perforated with a shaped Confianza Pro 12 wire (Asahi Intecc, Japan) (Figure 2H). The membrane was fenestrated with a 2.0 × 20 mm semi‐compliant balloon followed by kissing‐balloon dilatation. Due to persistent bleeding, optical coherence tomography (OCT) was performed, revealing malapposition of the covered stent in the LM, resulting in a type III endoleak. Culotte stenting was deemed necessary. Therefore, a third covered stent (Papyrus 3.0 × 15 mm) was implanted from the LM into the LCX (Figure 2I) followed by a proximal optimization technique (POT). The covered stent was fenestrated toward the LAD using the previously described technique with the Confianza Pro 12 wire (Figure 2J) and the procedure was completed with a final kissing‐balloon dilatation. The OCT imaging identified some damage of the membrane and a mild endoleak (Figure 2Q). However, due to only minimal residual bleeding (Ellis Type 1) we decided to conclude the procedure (Figure 2K/L).

Subsequently, the patient hemodynamically stabilized and could be weaned from va‐ECMO 48 h later. During percutaneous explantation of the ECMO a second‐look angiography was performed, demonstrating a good result without any residual bleeding (Figure 2M).

## 3 Discussion

CPs are a rare but serious complication in interventional cardiology. The severity of CPs is commonly classified using the Ellis classification (Table 1), with Type 3 representing the most severe form [3]. CP lead to significantly increased mortality rates [4, 5], also depending on the severity of the complication [6].

**Table 1:** Ellis classification of coronary perforations [3].

Ellis class	Definition
Type I	Extraluminal crater without extravasation
Type II	Pericardial or myocardial blush without contrast jet extravasation
Type III	Extravasation through frank (≥ 1 mm) perforation
Cavity spilling	Perforation into an anatomic cavity chamber, coronary sinus, and so on

An analysis of the United Kingdom interventional database from 2006 to 2013 by Kinnaird et al. found an incidence for CP during PCI of 0.33% [4]. They identified age, previous coronary artery bypass grafting (CABG), LM PCI, use of rotational atherectomy and presence of CTO as risk factors for CP. In an additional analysis of the same database, the incidence of CP during LM PCI was 0.9% [5]. In this cohort, female sex, number of stents, use of rotational atherectomy and presence of CTO were identified as risk factors for CP. Although the reported incidence is relatively small, these numbers show that CP is not an uncommon complication. Therefore, it is essential to be prepared for its occurrence by understanding the different treatment strategies and having the appropriate materials available.

While some Ellis type 1 and 2 CP can be managed conservatively, immediate balloon inflation is the most common strategy for all relevant CP in order to prevent cardiac tamponade. In the event of pericardial effusion, pericardiocentesis should be performed, preferably with autotransfusion. Sequential repositioning of the balloon may help to identify the exact location of the CP. While prolonged balloon occlusion is often sufficient for smaller CP, Ellis type 3 CP commonly require the use of additional methods [7]. Notably, the treatment strategy also depends on the cause and location of the CP. Coiling for example is a proven method for the treatment of distal wire perforations, whereas large vessel CP usually require the implantation of a covered stent. A commonly used method to safely exchange equipment while maintaining hemostasis is the so‐called *ping‐pong technique* [8]. This method uses the introduction of a second guide catheter in the same coronary artery to insert additional material, while balloon occlusion is preserved through the first catheter.

Management of CP is exceptionally challenging if it involves a bifurcation, especially the LM. Because a surgical approach is often not feasible due to limited accessibility and increased operative risk, an interventional approach is usually preferable [9]. On the other hand, the use of a covered stent inevitably results in side branch occlusion, thereby inducing ischemia corresponding to the size of the branch. As described in the two cases, fenestration of covered stents is a feasible approach for preserving a relevant side branch. In addition to our report, nine cases with successful covered coronary stent fenestration have been published so far (Table 2), which further confirms the general concept. The perforation of the membrane was performed with a stiff guidewire, supported by either a dual‐lumen microcatheter or an angled microcatheter. The most commonly used wires were the Hornet 14 (Boston Scientific, USA), Astato 20 (Asahi Intecc, Japan) and Confianza Pro 12. Visualization of the side branch, either with a radiopaque guidewire or a previously implanted stent, should be considered to guide the perforation of the membrane (Figures 1E and 2H/J). Subsequently, balloon dilatation is performed to restore perfusion of the side branch. The Papyrus was the most commonly used covered stent with this technique, but fenestration has also been performed using the BeGraft coronary stent (Bentley InnoMed, Germany) and the Graftmaster (Abbott vascular, USA). However, perforation of the latter two stent types seemed to have been more difficult. Regarding the Graftmaster, this might be due to the sandwich design of the stent, where the membrane is placed between two stainless steel stents. While perforation of the Papyrus stent seems to be easier, a potential limitation is the relative fragility of its membrane. Doost et al. described a recurrent perforation caused by a tear in the Papyrus membrane, which was visible on OCT imaging [15]. They speculated that fractured calcium resulting from previous intravascular lithotripsy, may have contributed to the stent failure. This possibility should therefore be considered, when sealing a CP with a covered stent fails, especially in heavily calcified vessels. Intracoronary imaging can be helpful in identifying this issue, as well as a stent malapposition, which is another common cause of unsuccessful CP treatment.

**Table 2:** Overview of published cases of covered stent fenestration (2014–2025).

References	Vessel involved	Bifurcation technique	Type of covered stent	Microcatheter for fenestration	Guidewire for fenestration
Holck et al. 2025 [10]	LAD, D2	Culotte Stenting with covered stents	Papyrus (Biotronik)	Sasuke dual lumen microcatheter (Asahi Intecc)	Halberd (Asahi Intecc)
Maznyzka et al. 2023 [9]	LM, LAD	Provisional stenting LM‐LAD	Papyrus (Biotronik)	SuperCross Angled‐Tip 120° (Teleflex)	Astato 20 (Asahi Intecc)
Allana et al. 2023 [11]	RCA, RPL, PDA	Covered stent RPL‐PDA over venous bypass (in‐stent stenosis)	Papyrus (Biotronik)	Antegrade from extraplaque space Microcatheter type not specified	Hornet 14 (Boston Scientific)
		Antegrade culotte Stenting RCA‐RPL‐PDA			
Colangelo et al. 2022 [12]	LM, LAD	Culotte Stenting LM‐LAD‐LCX with regular stents, Covered stent LM‐LAD	BeGraft coronary (Bentley InnoMed)	Antegrade (unsuccessful): SuperCross Angled‐Tip 90 (Teleflex)	Hornet 14 (Boston Scientific)
				Retrograde (successful over septal collateral):	
				Finecross (Terumo)	
Cilia et al. 2022	LM, LAD, LCX	Initially DK‐crush LM‐LAD‐LCX.	Papyrus (Biotronik)	90°‐angled microcatheter (no further specification)	Astato 20 (Asahi Intecc)
		Covered Stent LM‐LAD + regular Stent LCX in TAP‐technique			
Arbas‐Redondo et al 2021 [13]	LAD, D2	Provisional Stenting with covered stent to treat coronary aneurysm	Papyrus (Biotronik)	SuperCross Angled‐Tip 90° (Teleflex)	Hornet 14 (Boston Scientific)
Adusumalli et al. 2019 [14]	LM, LAD, LCX	Culotte Stenting LM‐LAD‐LCX with regular stents, Covered stent LM‐LCX	Papyrus (Biotronik)	Dual lumen microcatheter (Kaneka, Japan)	Astato 20 (Asahi Intecc)
					Confianza Pro 12 (Asahi Intecc)
Werner et al. 2017 [2]	LM, LAD	Provisional stenting LM‐LAD with covered stent due to ostial LM perforation	Papyrus (Biotronik)	FineDuo dual lumen catheter (Terumo)	Confianza Pro 12 (Asahi Intecc)
Taniguchi et al. 2014 [1]	LM, LAD	Provisional stenting LM‐LAD	Graftmaster (Abbott)	Crusade dual lumen micro catheter (Kaneka)	Unsuccessful:
					Gaia Third, Confianza, Confianza 12 (Asahi Intecc)
					Successful:
					Confianza 8‐20 (Asahi Intecc)


Abbreviations: D2, second diagonal branch; LAD, left anterior descending artery; LCX, left circumflex artery; LM, left main coronary artery; PDA, posterior descending artery; RCA, right coronary artery; RPL, posterolateral branch; TAP, T‐and‐protrusion.

In addition to the fenestration, a two‐stent technique may sometimes be necessary. For the treatment of relevant side branch stenosis, for example due to plaque‐shift, the use of a kissing balloon dilatation and/or conventional drug‐eluting stents are often sufficient after successful fenestration of the covered stent. However, in the case of persistent CP, the use of a second covered stent should be considered. This may be necessary even in cases where the decision has been made to sacrifice the side branch. As described in case 1, collateral flow may lead to an endoleak‐like situation, where fenestration followed by additional implantation of a covered stent is required to definitively treat the CP (Figure 1D). In this case, the TAP‐technique with two covered stents was used. Another possible approach is the culotte‐technique, as described in case 2. Holck et al. also published a case of culotte stenting of a bifurcation with two PK Papyrus stents [10], further demonstrating the feasibility of this method.

**Figure 3:**
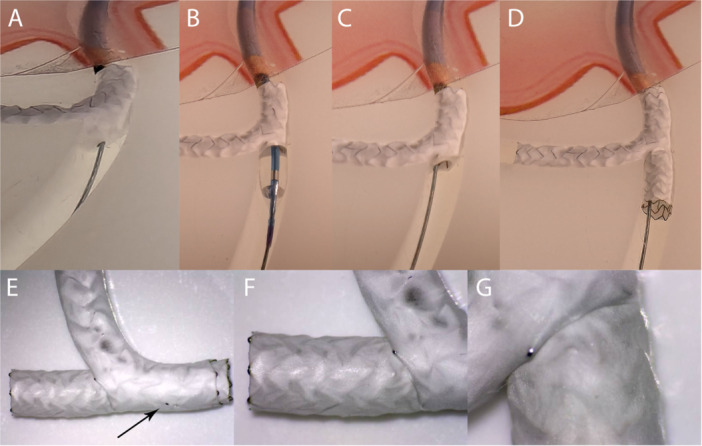
Bench test. (A) Perforation of the first stent membrane with a stiff guidewire (Confianza Pro 12). (B)/(C) Fenestration of the stent membrane with a non‐compliant balloon. The dilatation leads to a relatively round hole in the membrane, which would be sufficient to restore the circulation in this vessel. (D)/(E) Final result after culotte stenting. The arrow indicates a small wire perforation which occurred during the perforation attempt. (F)/(G) Close up of the seam between membranes of the two covered stents, which appear to form an adequate seal. [Color figure can be viewed at wileyonlinelibrary.com]

To visualize the interaction between the membranes of the two covered stents, we performed reverse culotte‐stenting with two Papyrus stents in a coronary bench model (Interventional Medical Device Solutions, The Netherlands). After implantation of the first stent into the LCX, the membrane was fenestrated toward the LAD using a Confianza Pro 12 guidewire (Figure 3A). This step was technically easy and feasible without support of a microcatheter. In the bench model, a small hole in the membrane resulting from the perforation attempts could be visualized (Figure 3E). This reaffirms that perforation and fenestration of a covered stent membrane is a feasible approach; however, cautious navigation of the stiff guidewire is essential. The use of a microcatheter to guide this procedure seems to be mandatory for accurate perforation, since an accidental perforation at the wrong site of the membrane may lead to an endoleak. The procedure was finalized with implantation of the second covered stent from the LM into the LAD (Figure 3D), fenestration toward the LCX with a stiff wire, kissing‐balloon inflation and final POT. Upon completion, the two membranes seemed to form a tight seal at the site where the second stent penetrated the first (Figure 3F/G).

Overall, several considerations must be taken into account before performing culotte stenting with covered stents. First, the technique requires two successive membrane perforation maneuvers, increasing the overall complexity of the procedure. Furthermore, the sequential occlusion of both affected vessels may lead to hemodynamic compromise, particularly during treatment of a LM bifurcation. Another very important consideration is the limited overexpansion capability of the covered stents. Currently there are only two covered stent types available, since the Graftmaster will be discontinued. The published maximum expansion diameters for the Papyrus are shown in Table 3. These stent characteristics are generally sufficient for the treatment of most bifurcations; however, they are not comparable to the expansion properties of contemporary DES. In contrast, the potential overexpansion capability of the BeGraft coronary stent has not been reported in the literature; therefore, it should be used with caution in this setting. A tear in the membrane caused by exceeding the stent's overexpansion capability—potentially facilitating recurrent bleeding—should be avoided. In case of large differences in vessel diameters, use of the TAP‐technique may be considered as an alternative to culotte stenting. Takagi et al. described an alternative technique for treatment of CP in the LM bifurcation using a jailed balloon in the LCX during implantation of a covered stent from the LM to the LAD [17], comparable to a bifurcation treatment with kissing stents. However, this approach was only possible because the CP was localized directly opposite the LCX ostium.

**Table 3:** Expansion diameters for the PK Papyrus (Biotronik).

Nominal stent diameter	Maximum stent expansion diameter (as published online [16])
ø 2.5–3.0 mm	3.5 mm
ø 3.5–4.0 mm	4.65 mm
ø 4.5–5.0 mm	5.63 mm

Another important general aspect is the limited deliverability of the covered stents. Furthermore, the increased rate of in‐stent restenosis is a well‐recognized limitation of these devices, regardless of the graft type used [18, 19]. The pathogenesis of this issue differs partially from regular DES. While restenosis on the edges of a covered stent is usually caused by neointimal hyperplasia, the middle of these stent grafts might have no neointimal coverage at all, even after 9 months [20, 21]. This may lead to thrombus formation which has been identified as the underlying cause of restenosis in some cases, so prolonged dual antiplatelet therapy should be considered. The implantation of a DES, instead of a covered stent, has been proposed as an alternative treatment for CP, to circumvent the issue of restenosis, or at least provide an antiproliferative outside layer for a covered stent [22]. There are, however, several limitations to this approach. Firstly, in our experience, a DES is rarely sufficient to treat an Ellis type 3 perforation. Additionally, the proposed antiproliferative outside layer of a DES might prevent the formation of neointimal hyperplasia after subsequent covered stent implantation, but cannot solve the issue of incomplete neointimal coverage. Furthermore, when used in a bifurcation setting, stent‐deformation due to kissing‐balloon dilatation or POT may lead to recurrence of the CP. Although the implantation of a DES might be sufficient in some cases, the drawbacks of another stent layer should also be considered, especially in a bifurcation. In general, the consideration of restenosis is secondary in the context of a bail‐out procedure intended to manage a potentially life‐threatening complication. A surgical approach remains the final option if interventional treatment is not feasible or fails.

## 4 Conclusions

Although rare, CP is a serious complication for which interventional cardiologists must be prepared, particularly in the context of complex coronary interventions. Fenestration of covered stents and the subsequent use of two‐stent techniques is a feasible method for managing CP localized at bifurcations. Operators should be familiar with the available treatment options and the necessary equipment must be readily accessible.

## Conflicts of Interest

The authors of this article have no relationship with the industry, relevant financial association, or other conflicts of interest to disclose. The coronary bench model was provided by BIOTRONIK, Germany.

## References

[1] N. Taniguchi , A. Takahashi , Y. Mizuguchi , T. Yamada , T. Hata , and S. Nakajima , “Successful Recanalization of a Left Circumflex Artery Jailed With a Polytetrafluoroethylene‐Covered Stent After Coronary Perforation During Stent Implantation in the Left Main Bifurcation,” Cardiovascular Intervention and Therapeutics 30, no. 1 (2015): 78–81, 10.1007/s12928-014-0254-8. 24557981

[2] G. S. Werner and W. H. Ahmed , “Fenestration of a Papyrus PK Covered Stent to Recover the Occluded Left Main Bifurcation After Sealing a Left Main Perforation During a CTO Procedure,” Cardiovascular Revascularization Medicine 18, no. 6 (2017): 41–44, 10.1016/j.carrev.2017.03.006. 28314672

[3] S. G. Ellis , S. Ajluni , A. Z. Arnold , et al., “Increased Coronary Perforation in the New Device Era. Incidence, Classification, Management, and Outcome,” Circulation 90, no. 6 (1994): 2725–2730, 10.1161/01.CIR.90.6.2725. 7994814

[4] T. Kinnaird , C. S. Kwok , E. Kontopantelis , et al., “Incidence, Determinants, and Outcomes of Coronary Perforation During Percutaneous Coronary Intervention in the United Kingdom Between 2006 and 2013: An Analysis of 527 121 Cases From the British Cardiovascular Intervention Society Database,” Circulation. Cardiovascular Interventions 9, no. 8 (2016): e003449, 10.1161/CIRCINTERVENTIONS.115.003449. 27486140

[5] H. I. Hussain , M. B. Protty , S. Gallagher , et al., “The Impact of Coronary Perforation in Percutaneous Interventions Involving the Left Main Stem Coronary Artery in the United Kingdom 2007–2014: Insights From the British Cardiovascular Intervention Society Database,” Catheter Cardiac Intervention 97, no. 2 (2021): 175–185, 10.1002/ccd.28933. 32333715

[6] A. J. Lansky , Y. Yang , Y. Khan , et al., “Treatment of Coronary Artery Perforations Complicating Percutaneous Coronary Intervention With a Polytetrafluoroethylene‐Covered Stent Graft,” American Journal of Cardiology 98, no. 3 (2006): 370–374, 10.1016/j.amjcard.2006.02.041. 16860026

[7] K. Meguro , H. Ohira , T. Nishikido , et al., “Outcome of Prolonged Balloon Inflation for the Management of Coronary Perforation,” Journal of Cardiology 61, no. 3 (2013): 206–209, 10.1016/j.jjcc.2012.11.007. 23380534

[8] Y. Ben‐Gal , G. Weisz , M. B. Collins , et al., “Dual Catheter Technique for the Treatment of Severe Coronary Artery Perforations,” Catheterization and Cardiovascular Interventions: Official Journal of the Society for Cardiac Angiography & Interventions 75, no. 5 (2010): 708–712, 10.1002/ccd.22331. 20049957

[9] A. Maznyczka , S. Arockiam , H. Bulluck , and A. Mozid , “Follow‐Up Optical Coherence Tomography to Evaluate Circumflex Ostium After Fenestration of Left Main Papyrus Covered Stent: A Case Report,” European Heart Journal ‐ Case Reports 7, no. 9 (2023): ytad415, 10.1093/ehjcr/ytad415. 37662583 PMC10473851

[10] E. N. Holck , L. N. Andreasen , E. K. Hansen , et al., “Complex Treatment of a Coronary Bifurcation Perforation With a Dual‐Covered Stent Strategy,” JACC. Case Reports 30, no. 7 (2025): 103382, 10.1016/j.jaccas.2025.103382. 40185573 PMC12046829

[11] S. S. Allana , J. Karacsonyi , S. Kostantinis , B. Simsek , A. Rempakos , and E. S. Brilakis , “Fenestration of a PK Papyrus Stent From an Extraplaque Space Into the True Lumen,” JACC. Cardiovascular Interventions 16, no. 9 (2023): 1109–1112, 10.1016/j.jcin.2023.02.033. 37164612

[12] S. Colangelo , A. Sardone , F. Colombo , G. Boccuzzi , and M. Iannaccone , “Retrograde Fenestration of Covered Stent After Left Main–Left Anterior Descending Perforation,” JACC. Cardiovascular Interventions 15, no. 21 (2022): 227, 10.1016/j.jcin.2022.07.048. 36357039

[13] E. Arbas‐Redondo , A. Jurado‐Román , S. Jiménez‐Valero , G. Galeote‐García , A. Gonzálvez‐García , and R. Moreno‐Gómez , “Acquired Coronary Aneurysm After Stent Implantation at a Bifurcation Excluded With a Papyrus Covered Stent Subsequently Fenestrated,” Cardiovascular Intervention and Therapeutics 37, no. 1 (2022): 215–216, 10.1007/s12928-021-00760-z. 33512648

[14] S. Adusumalli , N. Gaikwad , C. Raffel , and R. Dautov , “Treatment of Rotablation‐Induced Ostial Left Circumflex Perforation by Papyrus Covered Stent and Its Fenestration to Recover the Left Anterior Descending Artery During Chip Procedure,” Catheter Cardiac Intervention 93, no. 6 (2019): 331–336, 10.1002/ccd.28114. 30790419

[15] A. Doost , T. Mabote , R. Clugston , and A. R. Ihdayhid , “A Case of Covered Stent Failure in Sealing Up a Coronary Perforation Potentially Related to Intravascular Lithotripsy Treatment: Insights From Optical Coherence Tomography,” European Heart Journal ‐ Case Reports 6, no. 10 (2022): 1–5, 10.1093/ehjcr/ytac410. PMC9606236 36320378

[16] Biotronik AG PK Papyrus Brochure . Coronary Covered Coronary Stent System 2021.

[17] K. Takagi , K. Furui , I. Morishima , and H. Tsuboi , “Usefulness of the Jailed‐Balloon Technique in Percutaneous Intervention for Severe Coronary Perforation Involving Left Main Bifurcation,” JACC. Cardiovascular Interventions 10, no. 22 (2017): 209, 10.1016/j.jcin.2017.07.050. 29102580

[18] V. Nagaraja , K. Schwarz , S. Moss , C. S. Kwok , and M. Gunning , “Outcomes of Patients Who Undergo Percutaneous Coronary Intervention With Covered Stents for Coronary Perforation: A Systematic Review and Pooled Analysis of Data,” Catheterization and Cardiovascular Interventions: Official Journal of the Society for Cardiac Angiography & Interventions 96, no. 7 (2020): 1360–1366, 10.1002/ccd.28646. 31850685

[19] M. Hernández‐Enríquez , L. Belle , H. Madiot , et al., “Use and Outcomes of the PK Papyrus Covered Stent in France: SOS PK Papyrus Registry,” Catheterization and Cardiovascular Interventions: Official Journal of the Society for Cardiac Angiography & Interventions 98, no. 5 (2021): 874–881, 10.1002/ccd.29328. 33085150

[20] K. Kongoji , Y. Ishibashi , N. Kotoku , et al., “Angioscopic and Optical Coherence Tomographic Evaluation of Neointimal Coverage: 9 Months After Expandable Polyterafluoroethylene Covered Stent Implantation,” Heart and Vessels 32, no. 6 (2017): 777–779, 10.1007/s00380-017-0964-9. 28289840 PMC5446842

[21] Q. Yang , W. Liu , F. Fang , C.‐M. Yu , and Y.‐J. Zhou , “In‐Stent Restenosis in a Polytetrafluoroethylene Covered Stent Combined With Drug Eluting Stents: Potential Pathogenesis Revealed by Optical Coherence Tomography,” International Journal of Cardiology 198 (2015): 42–44, 10.1016/j.ijcard.2015.06.086. 26151708

[22] H. Munirwan , T. F. Hadi , A. Purnawarman , M. H. Latief , W. Wattanasiriporn , and T. Yusrizal , “Nearly Catastrophe Coronary Perforation: Is It Second Drug‐Eluting Stent Effective?,” Narra J 4, no. 1 (2024): e637, 10.52225/narra.v4i1.637. 38798874 PMC11125394

